# Risk factors for postoperative cerebrospinal fluid leakage after transsphenoidal surgery for pituitary adenoma: a meta-analysis and systematic review

**DOI:** 10.1186/s12883-021-02440-0

**Published:** 2021-10-27

**Authors:** Zhihuan Zhou, Feifei Zuo, Xiaoqun Chen, Qinqin Zhao, Mengna Luo, Xiaobing Jiang, Yuyu Duan

**Affiliations:** 1 State Key Laboratory of Oncology in South China, Collaborative Innovation Center for Cancer Medicine, and Guangdong Key Laboratory of Nasopharyngeal Carcinoma Diagnosis and Therapy, Sun Yat-sen University Cancer Center, Guangzhou, Guangdong PR China; 2 Department of Neurosurgery, Sun Yat-sen University Cancer Center, Guangzhou, Guangdong PR China; 3 Department of Nasopharyngeal Carcinoma, Sun Yat-sen University Cancer Center, 651 Dongfeng Road East, Guangzhou, 510060 PR China

**Keywords:** Cerebrospinal fluid leakage, Transsphenoidal surgery, Pituitary adenoma, Meta-analysis, Systematic review

## Abstract

**Objective:**

Postoperative cerebrospinal fluid (CSF) leakage represents a challenge even for experienced pituitary surgeons. We aimed to quantitatively synthesize data from studies regarding the risk factors for postoperative CSF leakage after transsphenoidal surgery (TSS) for pituitary adenoma (PA).

**Methods:**

PubMed, Web of Science, The Cochrane Library, Embase, China National Knowledge Infrastructure (CNKI), Wanfang database, and VIP database were searched for case–control and cohort studies, focusing on the risk factors associated with postoperative CSF leakage after TSS for PA. Pooled odds ratios (ORs) and 95% confidence intervals were calculated to determine the risk factors.

**Results:**

A total of 34 case–control and cohort studies involving a total of 9,144 patients with PA were included in this systematic review. The overall rate of postoperative CSF leakage after TSS for PA was 5.6%. Tumor size, adenoma consistency, revision surgery, and intraoperative CSF leakage were independent risk factors for postoperative CSF leakage (ORs, 3.18–6.33). By contrast, the endoscopic approach showed a slight protective benefit compared with the microscopic approach in TSS (OR, 0.69).

**Conclusions:**

This review provides a comprehensive overview of the quality of the evidence base, informing clinical staff of the importance of screening risk factors for postoperative CSF leakage after TSS for PA. More attention should be paid to PA patients at high risk for CSF leakage after TSS to reduce complications and improve prognosis.

**Supplementary Information:**

The online version contains supplementary material available at 10.1186/s12883-021-02440-0.

## Introduction

Pituitary adenomas (PAs) are benign neoplasms that represent the most common type of pituitary disorder [1]. A series of clinical case studies have reported a prevalence for PA among community-dwelling adults ranging from 1 in 865 to 1 in 2,688 [2]. The goals of PA surgery include the complete removal of the adenomas, the correction of hormonal hypersecretion, the retention of pituitary function, and the reduced risk of tumor recurrence [3, 4]. Compared with transcranial surgery, transsphenoidal surgery (TSS) is advantageous because it does not require brain retraction, resulting in fewer complications, shorter hospital stays, and better patient comfort [5]. With the evolution of imaging and surgical techniques, TSS has become an effective and preferred surgical approach for most PAs [4], associated with an extremely low mortality rate [3].

Although TSS for PA is considered safe [6], possible complications can occur. Postoperative cerebrospinal fluid (CSF) leakage represents a characteristic and potentially severe side effect [7] and remains a challenge even for experienced pituitary surgeons. Previous studies have reported that the incidence of CSF leakage after TSS ranged from 0.5% to 15% [7]. A national survey of complications after TSS found that although various repair methods have been developed, the incidence of postoperative CSF leakage remained high (3.9%) [8]. If not treated properly, postoperative CSF leakage can lead to serious consequences, such as headache, incision infections, meningitis, and even encephalopyosis [9], which can be life-threatening, resulting in patients with enormous economic and psychological burdens [10].

Elucidating the risk factors associated with CSF leakage after TSS is important and helpful for patients with PA. The identification of these factors may permit the implementation of strategies to reduce postoperative complications. However, limited attention has been paid to factors associated with such CSF leaks. Some differences have been reported among the results of previous studies on risk factors, which may be due to different inclusion criteria and small sample sizes. No comprehensive meta-analysis has been performed to examine the risk factors for CSF leakage after TSS. Therefore, the purpose of our article was to systematically review the potential risk factors associated with the development of postoperative CSF leaks after TSS in patients with PA.

## Methods

### Data sources

A search was performed in PubMed, Web of Science, The Cochrane Library, Embase, China National Knowledge Infrastructure (CNKI), Wanfang database, and VIP database to identify articles published from database inception to December 2020. We used a search strategy that included truncated free text and exploded medical subject heading (MeSH) terms relevant to “pituitary adenoma,” “postoperative cerebrospinal fluid leak,” “transsphenoidal surgery,” or “risk factors.” For example, the search details for PubMed/Medline were as follows: (((“pituitary adenoma”[Title/Abstract] OR “pituitary adenoma”[All Fields] OR “pituitary tumor”[Title/Abstract] OR “pituitary tumor”[All Fields]) AND (“cerebrospinal fluid leak”[MeSH Terms] OR (“cerebrospinal”[All Fields] AND “fluid”[All Fields] AND “leak”[All Fields]) OR “cerebrospinal fluid leak”[All Fields])) AND (“transnasal”[Title/Abstract] OR “transnasal approach”[Title/Abstract] OR “transnasal approach”[All Fields] OR “transsphenoidal surgery”[Title/Abstract] OR “transsphenoidal surgery”[All Fields]) AND “risk factor”). In addition, manual searches were conducted of the reference sections of retrieved articles to identify additional published work relevant to this study.

### Study selection

All retrieved studies were reviewed for inclusion based on the following criteria: 1) patients who underwent TSS and were pathologically diagnosed with PA according to postoperative immunocytology; 2) studies mentioning CSF leakage after TSS; and 3) prospective or retrospective studies.

The exclusion criteria were as follows: 1) spontaneous CSF leakage; 2) case reports, reviews, letters, or dissertation; 3) articles in other languages than English and Chinese; 4) reported only duplicate data; 5) no data regarding postoperative CSF leakage; 6) cadaver or animal studies; and 7) articles with incomplete information or incomplete data.

Two reviewers independently screened the titles and abstracts of all articles for eligibility. The screening results from the two reviewers were compared, and disagreements were discussed to reach a consensus. Next, full-text reviews and data extraction were independently performed by the same two reviewers, and the results were again compared and discussed to reach an agreement. A third reviewer was consulted to assist in the resolution of unresolved discrepancies between reviewers at any stage in the article selection process.

### Data extraction and quality assessment

Two authors independently extracted data from the selected articles using a standard data extraction form. Data included the following study items: publication year, first author, study design, sample size, population characteristics, number of CSF leaks after TSS, and associated risk factors.

Two authors independently assessed the quality of all included studies using the Newcastle–Ottawa Scale (NOS) [11]. The NOS assesses quality in terms of the selection of PA patients; the comparability of study groups, if applicable; and outcome assessments. Differences in quality assessments between the two authors were resolved discussion until consensus was reached. Studies with an NOS score ≥ 6 that included appropriate statistical analyses were deemed to have high methodological quality.

### Statistical analyses

Review Manager software (RevMan 5.3; Cochrane Collaboration, Oxford, United Kingdom) was used to perform statistical analyses. For each of the dependent factors, effect sizes were calculated separately. For each dependent variable, an overall effect size was calculated by weighting all the effect sizes calculated for each individual study according to relative sample sizes [12]. To test whether the variability in effect sizes exceeded what could be expected from sampling error alone, I^2^ tests for heterogeneity were conducted. When the I^2^ value was less than 50%, indicating low heterogeneity, a fixed-effects analysis was used to estimate the assumed common effect. When the I^2^ value was 50% or above, indicating increased heterogeneity, a random-effects analysis was used to estimate the mean distribution of effects across all studies, yielding wider confidence intervals for the combined effect size [13]. In addition, a sensitivity analysis was performed by eliminating each included study one at a time when the heterogeneity was high (I^2^ ≥ 50%). The odds ratios (ORs) and corresponding 95% confidence intervals (95% CIs) were calculated for categorical variables, based on the number of postoperative CSF leakage groups and the total sample size for each risk factor. Funnel plots were used to assess publication bias and were generated for all factors that were identified in more than 10 studies included in the present study [13].

## Results

### Literature search and study characteristics

**Fig. 1:**
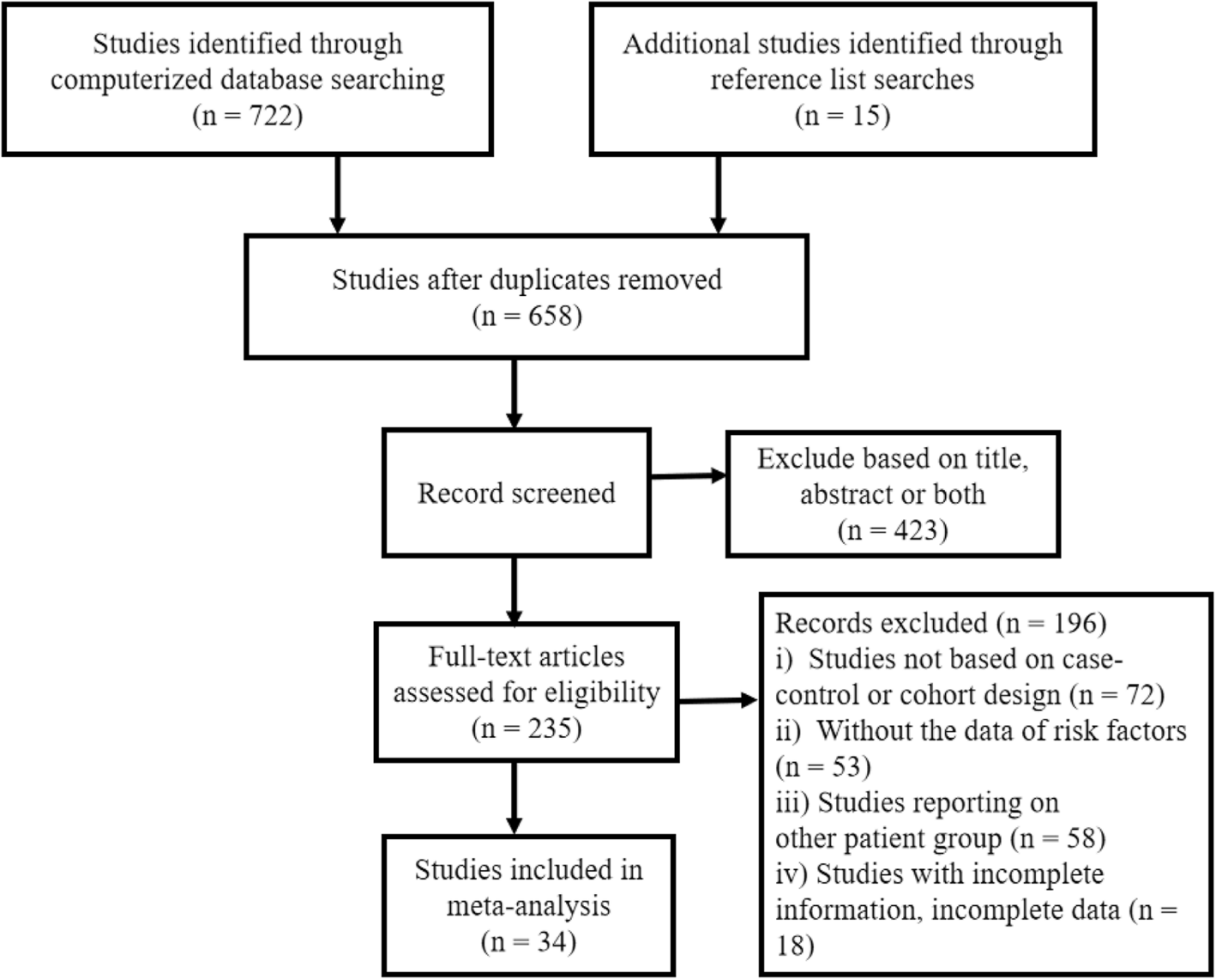
Flow diagram of study search and criteria application

A total of 14 case–control studies and 20 cohort studies were included in the present analysis. A flow diagram of the selection and exclusion processes, together with the respective justifications for exclusion, is shown in Fig. 1. After the application of inclusion and exclusion criteria, 34 articles were included in our quantitative analysis, and the characteristics of the 34 studies are summarized in Table 1. A total of 9,144 patients were enrolled in the meta-analysis, including 511 (5.6%) who were diagnosed with postoperative CSF leakage.

**Table 1:** Baseline characteristics of included studies

Study (first author)	Publication year	Study type	No. of cases with postop CSF leakage	Total no. of Patients	Risk factors	NOS score
Xu [14]	2013	case–control study	64	1641	3, 6	6
Cheng [15]	2014	case–control study	9	129	1, 2, 3, 4, 6	6
Tang [16]	2018	cohort study	3	120	4	6
Zhao [17]	2020	cohort study	40	158	5	8
Li [18]	2016	cohort study	10	162	5	8
Liu [19]	2020	case–control study	25	194	1, 2, 4, 6	8
Tian [20]	2018	case–control study	29	1063	1, 3, 4, 6	8
Yin [21]	2014	cohort study	6	81	5	8
Wang [22]	2018	case–control study	11	112	4, 6	8
Zhang [23]	2017	case–control study	10	114	1, 4, 6	8
Huang [24]	2018	case–control study	33	270	1, 2, 4, 6	8
Ding [25]	2019	cohort study	22	251	5	9
Liu [26]	2014	cohort study	2	64	5	8
He [27]	2015	cohort study	2	47	5	8
Wang [28]	2011	cohort study	1	40	5	8
Yang [29]	2017	cohort study	13	240	5	8
Wangx [30]	2018	cohort study	11	64	5	8
Zhou [31]	2015	cohort study	15	116	5	8
Liu [32]	2017	cohort study	4	116	5	8
Chen [33]	2012	case–control study	25	180	1, 2, 3	6
Agam [34]	2018	cohort study	30	1153	5	6
Fujimoto [35]	2017	case–control study	8	162	1, 4, 6	8
Gao [36]	2016	cohort study	11	105	5	8
Guvenc [38]	2016	cohort study	7	94	5	8
Han [9]	2008	case–control study	26	592	1, 2, 3, 4	8
Karppinen [39]	2015	cohort study	6	185	5	8
Liu [40]	2019	cohort study	12	189	6	9
Mehta [41]	2012	cohort study	8	158	6	8
Riesgo [37]	2019	case–control study	7	302	3, 4	6
Xue [42]	2020	case–control study	13	216	4	6
Mansy [43]	2010	cohort study	8	200	6	6
Zhangc [10]	2017	case–control study	13	474	4, 6	7
Dallapiazza [44]	2014	cohort study	9	99	5	8
Thawani [45]	2016	case–control study	21	203	4, 6	9

Risk Factors: 1. Tumor size 2. Consistency of adenoma 3. Revision surgery 4. Intraoperative CSF 5. Operation method 6. Other factors: Sex, functional adenoma type, resection rate or perioperative lumbar drainage

*CSF* cerebrospinal fluid, *NOS* Newcastle–Ottawa Scale

## Risk factors for postoperative CSF leakage

### Tumor size

**Fig. 2:**
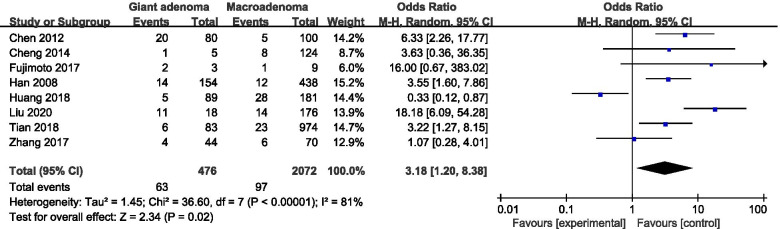
Forest plot of postoperative CSF leakage according to tumor size

A total of eight studies reported differences in the incidence of postoperative CSF leakage among patients with different tumor sizes (Fig. 2). The analysis of postoperative CSF leakage across tumor sizes revealed that giant adenoma was associated with a significantly increased risk of postoperative CSF leakage compared with macroadenoma or microadenoma (pooled OR: 3.18, 95% CI: 1.20–8.38). The sensitivity analysis suggested that when Huang’s study [24] was excluded, the result (pooled OR: 4.57, 95% CI: 2.31–9.05) was consistent with the overall result without excluding any studies. However, the heterogeneity remained high (I^2^ = 53%, *P* = 0.05).

### Consistency of adenoma

**Fig. 3:**
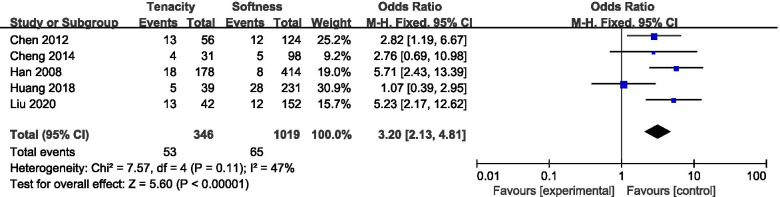
Forest plot of postoperative CSF leakage according to adenoma consistency

A total of five studies reported differences in the incidence of postoperative CSF leakage depending on the adenoma consistency (Fig. 3). The meta-analysis revealed that a hard tumor was associated with a significantly increased risk of postoperative CSF leakage compared with soft tumors (pooled OR: 3.20, 95% CI: 2.13–4.81).

### Primary vs. revision surgery

**Fig. 4:**
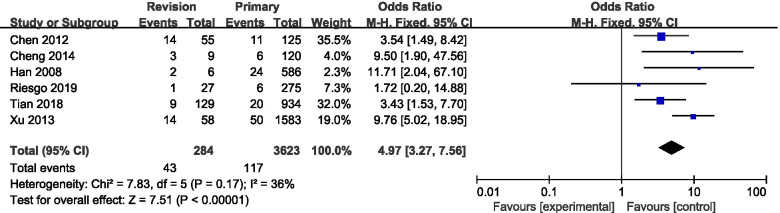
Forest plot of postoperative CSF leakage for revision surgery

A total of six studies provided data regarding the relationship between revision surgery and postoperative CSF leakage (Fig. 4). The meta-analysis revealed that revision surgery was associated with a significantly increased risk of postoperative CSF leakage compared with primary surgery (pooled OR: 4.97, 95% CI: 3.27–7.56).

### CSF during the operation (intraoperative CSF)

**Fig. 5:**
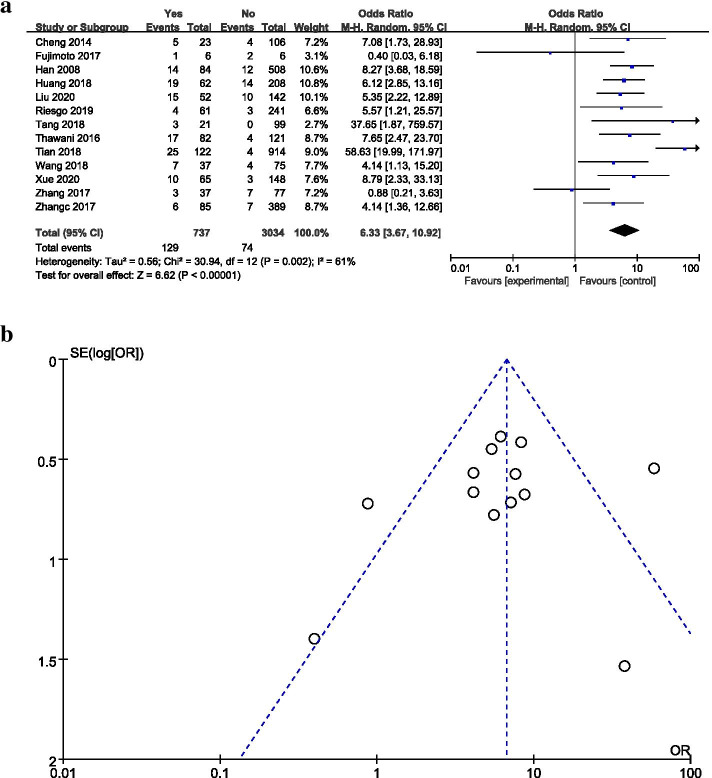
**a** Forest plot of postoperative CSF leakage according to the occurrence of intraoperative CSF leakage. **b** Funnel plot of postoperative CSF leakage for the occurrence of intraoperative CSF leakage

A total of 13 studies provided data regarding the relationship between intraoperative CSF leakage and postoperative CSF leakage (Fig. 5a). The meta-analysis revealed that intraoperative CSF leakage was associated with a significantly increased risk of postoperative CSF leakage (pooled OR: 6.33, 95% CI: 3.67–10.92) relative to no CSF leakage. The sensitivity analysis suggested that when Tian’s study [20] was excluded, the results remained stable (pooled OR: 5.33, 95% CI: 3.60–7.90), and the heterogeneity was reduced (I^2^ = 21%, *P* > 0.05). Through discussion among the researchers, Tian’s study was determined to meet the inclusion criteria and was retained. Using a funnel plot, most studies were found to be distributed in the center and top of the plot, indicating little publication bias (Fig. 5b).

### Operation method

**Fig. 6:**
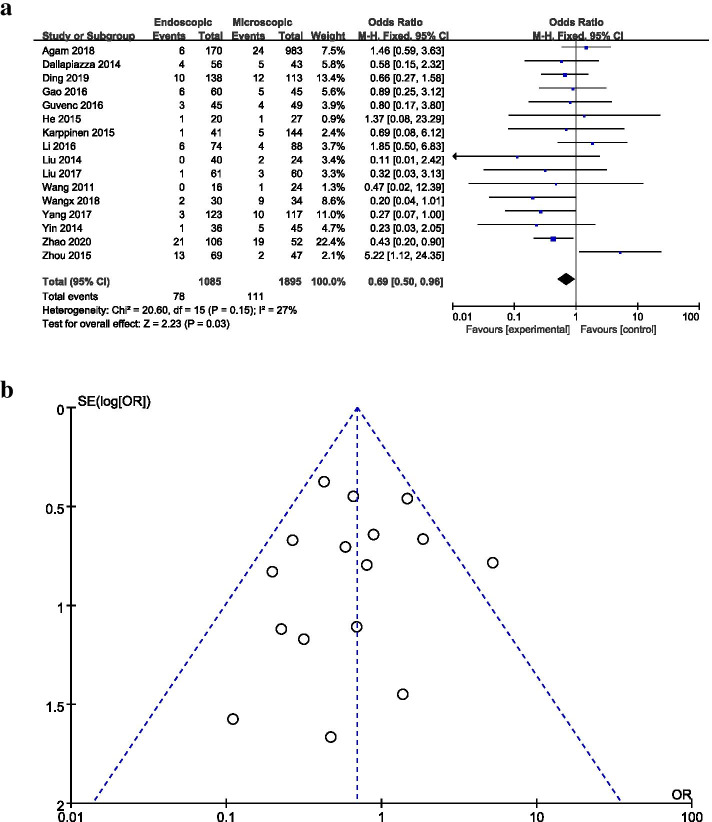
**a** Forest plot of postoperative CSF leakage according to the operation method. **b** Funnel plot of postoperative CSF leakage based on Operation method

A total of 16 studies reported differences in the incidence of postoperative CSF leakage between the endoscopic approach and the microscopic approach for TSS (Fig. 6a). The meta-analysis revealed that the endoscopic approach was associated with a significantly reduced risk of postoperative CSF leakage compared with the microscopic approach (pooled OR: 0.69, 95% CI: 0.50–0.96). In the generated funnel plot, most studies were distributed in the center and top of the plot, indicating little publication bias (Fig. 6b).

### Other factors

The meta-analysis suggested no significant difference in the incidence of postoperative CSF leakage across sexes (pooled OR: 1.78, 95% CI: 1.00–3.14) or functional adenoma types (pooled OR: 1.32, 95% CI: 0.84–2.05). Similarly, total resection (pooled OR: 0.77, 95% CI: 0.21–2.80) and no perioperative lumbar drainage (pooled OR: 0.61, 95% CI: 0.18–2.11) are not risk factors for postoperative CSF leakage. We have included these non-significant factors analyzed and the related diagrams in a “Supplementary Materials”.

## Discussion

This review quantitatively synthesized the currently available evidence on the prevalence and risk factors associated with the incidence of postoperative CSF leakage after TSS in 34 studies, which included 9,144 participants with PA, to demonstrate a pooled global prevalence of 5.6%. We evaluated the relationships between these nine factors and the risk of postoperative CSF leakage. Our data indicated that tumor size, adenoma consistency, revision surgery, and intraoperative CSF leakage were independent risk factors for postoperative CSF leakage. By contrast, the use of an endoscopic approach showed a significant protective benefit compared with the microscopic approach in TSS, whereas sex, functional adenoma type, resection rate, and perioperative LD were not found to be related to the occurrence of postoperative CSF leakage.

The effect of tumor size on postoperative CSF leakage is under debate, with some studies showing that patients with giant adenoma are more likely to experience postoperative CSF leakage than those with macroadenoma or microadenoma [9, 10]. In contrast, Shiley et al. [46] and Nishioka et al. [47] found that CSF leakage was more common among patients with microadenomas. In addition, Chi et al. [48] reported that tumor size was not associated with postoperative CSF leakage. Han et al. [9] indicated that large pituitary tumors commonly expand the sella and erode regions adjacent to the meninges, resulting in the attenuation of these barriers to the CSF space. Moreover, larger pituitary tumors result in the wider invasion of adjacent tissues, resulting in a greater extent of resection, which may increase the risk of CSF leakage after TSS [19]. In our study, tumor size was identified as a risk factor for postoperative CSF leakage, with high heterogeneity. Therefore, more high-quality research is necessary to better explore the effects of tumor size on postoperative CSF leakage occurrence.

The current analysis showed that adenoma consistency was associated with the occurrence of postoperative CSF leakage. Most PAs have a soft texture that can easily be resected with curettage and suction [49]. Hard (fibrous) tumors account for approximately 5%–13% of PA and are difficult to separate from critical structures, often requiring removal using sharp dissection or laser techniques [9, 49]. Therefore, the consistency of an adenoma affects its resection success [50]. In our meta-analysis, compared with soft tumors, firm pituitary tumors were found to be associated with a greater risk of CSF leakage after TSS, with crude ORs of 2.13–4.81. Therefore, the PA consistency must be assessed using reliable imaging techniques (such as magnetic resonance imaging, MRI) before performing TSS, which may help surgeons to better plan an appropriate operative strategy and reduce the risk of surgery.

A strong association was observed between revision surgery and an increased risk of postoperative CSF leakage, which is consistent with the findings of a previous study [9, 46, 47]. Shiley et al. [46] found that the incidence of postoperative CSF leakage after revision surgery was significantly higher than that after primary surgery (14.6% vs. 4.0%, P = 0.010) in a study examining 235 patients undergoing TSS. The first procedure can create scar tissue that adheres to the arachnoid space and diaphragm [51]. The revision surgery is made more difficult by the presence of adhesions, tissue fibrosis, abnormal vascular hyperplasia, and distorted anatomy, which can increase the complexity of the dissection [20]. Moreover, revision surgery requires the removal of residual tumors that were not easily detected or resectable during the prior surgery, which typically requires a more aggressive dissection approach along the sellar diaphragm, increasing the risk of postoperative CSF leakage [20].

In our results, patients with an intraoperative CSF leak were 6.33 times more likely to experience postoperative CSF leakage than patients without intraoperative CSF leakage. Seiler et al. [52] found that the occurrence of postoperative CSF leakage was six times as common among cases that reported intraoperative CSF leakage compared with those that did not. Similar results were observed in a retrospective analysis, which showed a significant difference in the rates of postoperative CSF leakage between patients who experienced an intraoperative CSF leak and those who did not (16.7% vs. 2.3%) [9]. Not surprisingly, intraoperative CSF leakage was correlated with the risk of postoperative CSF leakage, which may be due to the incomplete repair of intraoperative CSF leakage [20]. The unidentified or delayed development of intraoperative CSF leaks is also an equally important source of postoperative CSF leakage as the failure to employ effective CSF leak repair methods [53]. In addition, repair materials may shift or fall off due to postoperative actions that increase intracranial pressure (such as sneezing and constipation), resulting in incomplete leakage closure. Sarita et al. [54] found that chronic cough was one of the primary contributing factors to the failure to resolve an intraoperative leak, leading to the postoperative recurrence of the leak. Our findings suggested that patients with intraoperative CSF leaks may warrant more aggressive management to prevent the development of postoperative leaks, and correct behavior education is also important for the management of postoperative CSF leakage.

PAs with high levels of invasiveness are more likely to invade the peritumoral tissues, such as the cavernous sinus and the internal carotid artery, which may increase the difficulty of TSS. To completely remove the tumor, the surgeon may overstretch the sellar septum and dura mater, which increases the risk of CSF leakage [14]. In addition, the arachnoid membrane at the sellar diaphragmatic foramen can be easily damaged when most of the tissue around the PA is removed, resulting in CSF leakage. In our meta-analysis, few studies focused on the effects of invasiveness of PA on the occurrence of postoperative CSF leakage; therefore, we did not confirm whether this variable may serve as a clinically useful marker of an individual’s propensity toward postoperative CSF leakage. However, in cases of PA with high invasiveness, more attention should be given to local anatomy and surgical skill [55]. To prevent the occurrence of CSF leakage during TSS, the PA should be removed according to appropriate procedures, and the traction of the peritumoral tissues should be reduced as much as possible to avoid causing damage to the sellar septum and dura mater.

The current study found a mild association between the performance of endoscopic TSS and a reduced risk of postoperative CSF leakage, with crude ORs of 0.50–0.96. A meta-analysis of 23 observational studies conducted by Li et al. [56] previously showed that endoscopic TSS had no significant effect on the risk of CSF leakage compared with microscopic TSS. However, the data analyzed by Li et al. [56] cannot be directly compared with our series because their results were not limited to postoperative CSF leakage and encompassed all pituitary pathology. In addition, relatively few complications were reported. Compared with the microscopic approach, the endoscopic approach has many advantages. It is easier, requires a less traumatic entry into the sphenoid sinus, and enables wide and close views of the tumor, allowing for increased tumor resection [38]. By contrast, some surgeons believe that the endoscopic approach decreases stereoscopic visualization and decreases the ability to use their instruments [57], which may account for the lack of significant difference observed for the incidence of CSF leakage between these two surgical techniques in some studies [34, 38]. In our study, the endoscopic approach showed a minimally protective benefit for reducing postoperative CSF leakage compared with the microscopic approach. Surgeons must be specially trained for an endoscopic approach [38], and the surgeon’s learning curve was found to be associated with the occurrence of postoperative CSF leaks [58]. The results of endoscopic TSS may improve with the experience of individual surgeons. Therefore, further research remains necessary to explore the potentially protective role of using the endoscopic approach to prevent the occurrence of postoperative CSF leakage.

Although sex, aging, and body mass index (BMI) were reportedly associated with postoperative CSF leakage [58], the current evidence could not be used to determine whether these demographic factors were risk factors. A multi-institutional study of patients undergoing endoscopic PA showed that the risk of postoperative CSF leakage in female patients was 2.4 times higher than that in male patients [59]. However, Zhang et al. [23] and Tian et al. [20] have shown that the female sex of patients had no effect on the occurrence of postoperative CSF leakage. Whether sex is a risk factor for postoperative CSF leakage requires further investigation. Relatively few studies have examined the impacts of aging and BMI on postoperative CSF leakage in PA patients undergoing TSS, and various definitions of aging and BMI were used in these studies, making the meta-analysis of these two factors difficult to perform. Caitlin et al. [59] found that younger patients (<64 years) had a higher risk of postoperative CSF leakage than those older than 64 years. In two retrospective analyses, no correlation was observed between average patient age and the occurrence of postoperative CSF leakage [46, 47]. Several studies showed that an elevated BMI was an independent predictor of postoperative CSF leakage after TSS [59, 60]. This association might be due to the increased intra-abdominal pressure associated with higher BMI [58]. A moderate level of physical activity (e.g., 150 minutes of moderate aerobic exercise) for BMI reduction may be helpful [61]. In addition, more studies remain necessary to quantify the effects of aging and BMI on the occurrence of postoperative CSF leakage in PA patients undergoing TSS.

The present meta-analysis could not determine whether the functional adenoma type was associated with an increased risk of postoperative CSF leakage in PA patients undergoing TSS. Two studies found that postoperative CSF leakage was more common in adrenocorticotropic hormone (ACTH)-producing adenomas [46, 47], which is likely due to ACTH adenomas often not appearing localized on preoperative imaging and requiring a more aggressive resection approach [46, 47]. However, only a few events of postoperative CSF leakage were reported in these studies, and the conclusions were prone to bias. Han et al. [9] found that the postoperative leakage rates associated with follicle-stimulating hormone (FSH) adenomas were higher than those for other tumor types. By contrast, Tian et al. [20] and Wang et al. [22] found that the functional adenoma types were not a significant risk factor for postoperative CSF leakage; therefore, this inconsistency requires further study.

Whether the resection rate is a risk factor for postoperative CSF leakage remains controversial. A retrospective analysis of 1,641 patients with PA undergoing TSS showed that the incidence of postoperative CSF leakage in patients with total resection was significantly higher than that among patients with partial or subtotal resections [14]. The mechanism underlying this association may be that tumors that undergo total resection, especially for giant or invasive adenomas, experience more severe dural stretch, increasing the risk of postoperative CSF leakage [14]. The literature exploring the resection rate remains scarce and reported results have been conflicting and highly heterogeneous. Further studies must be performed to confirm the relationship between the resection rate and the occurrence of postoperative CSF leakage.

The use of LD remains under debate. In a meta-analysis [5], LD had an OR 1.13 for reducing the occurrence of postoperative CSF leakage after endonasal endoscopic skull base surgery. Gautam et al. [41] posited that preoperative or intraoperative LD could reduce tension on the arachnoid caused by the expansion of a pituitary macroadenoma, preventing the potential exposure of the arachnoid to intraoperative injury and reducing the risk of intraoperative CSF leakage. However, Jung et al. [5] suggested that these results should be interpreted carefully because surgeons tend to perform LD in high-risk patients or when the surgeon feels that the reconstruction was not completely successful. A prospective study of 114 TSS procedures for pituitary macroadenoma indicated that LD reduced the rate of intraoperative CSF leakage from 41 to 5% (p < 0.001), but the rate of postoperative CSF leakage remained similar (5 vs. 5%). In addition, some researchers have stated that the presence of the drain may mask the earlier detection of a CSF leak [43]. Therefore, whether LD reduces the risk of postoperative CSF leakage after TSS remains inconclusive given the current evidence.

The acknowledged limitations of this study should be mentioned. First, unpublished articles were not included in this systematic review, likely contributing to publication bias. Second, a lack of randomized control trials (RCTs) examining the occurrence of postoperative CSF leakage exists, which may reduce the reliability of the results of our study. Further studies using RCT designs may enrich and substantiate our results. Moreover, there were many kinds of fillers, such as gelfoam, absorbable hemostatic gauze, autologous fat, pedicled vascularized flap and so on. A specialized mesh meta-analysis is better used to compare which closure techniques can reduce the risk of CSF leakage. Despite these limitations, this study represents the first known meta-analysis to examine the risk factors associated with postoperative CSF leakage after TSS in PA patients.

This study makes a significant contribution to clinical practice because our findings can focus clinical medical workers’ attention on the occurrence of postoperative CSF leakage after TSS in PA patients. We have shown that which factors are more likely to cause CSF leakage in these patients for clinicians, especially neurosurgeons. When operating on PA patients with high-risk CSF leakage, surgeons should take some targeted strategies, such as selecting appropriate surgical methods and resection techniques. In addition, clinical nurses should strengthen observation of high-risk patients in postoperative nursing, (such as more frequent ward rounds), to achieve early detection and treatment of CSF leakage.

## Conclusion

This review provides a comprehensive overview of the quality of the evidence base to inform clinical staff of the importance of screening risk factors for postoperative CSF leakage after TSS for PA. The risk factors associated with postoperative CSF leakage after TSS include tumor size, adenoma consistency, revision surgery, and intraoperative CSF leakage. However, the use of the endoscopic approach showed a significant protective benefit compared with the microscopic approach in TSS, whereas sex, adenoma functional type, the resection rate, and perioperative LD were not identified as significant risk factors for CSF leakage in this meta-analysis. More attention should be paid to PA patients with high risks of CSF leakage after TSS to reduce complications and improve prognosis.

## Supplementary Information

**Additional file 1.**

## Data Availability

Not applicable.
