# Supplementary material for: Risk factors for postoperative cerebrospinal fluid leakage after transsphenoidal surgery for pituitary adenoma: a meta-analysis and systematic review
Source: BMC Neurol. 2021 Oct 27;21:417. doi: 10.1186/s12883-021-02440-0 (PMC8555154; doi:10.1186/s12883-021-02440-0)
Supplement: Supplementary file 1 — Additional file 1. [file 12883_2021_2440_MOESM1_ESM.docx]

**Sex**

A total of seven studies provided data on the relationship between sex and postoperative CSF leakage (Fig. 7). Meta-analysis suggested no significant difference in the incidence of postoperative CSF leakage across sexes (pooled OR: 1.78, 95% CI: 1.00–3.14).


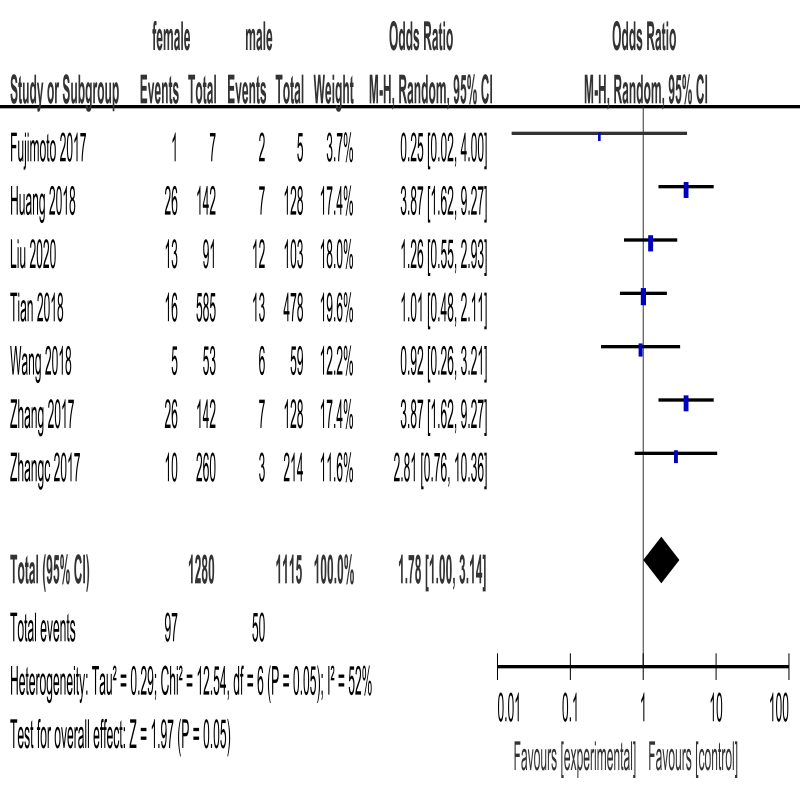


**Figure 7. Forest plot of postoperative CSF leakage according to sex**

**Functional adenoma type**

A total of five studies reported differences in the incidence of postoperative CSF leakage according to the functionality of the adenoma (Fig. 8). Meta-analysis suggested no significant difference in the incidence of postoperative CSF leakage across functional adenoma types (pooled OR: 1.32, 95% CI: 0.84–2.05).


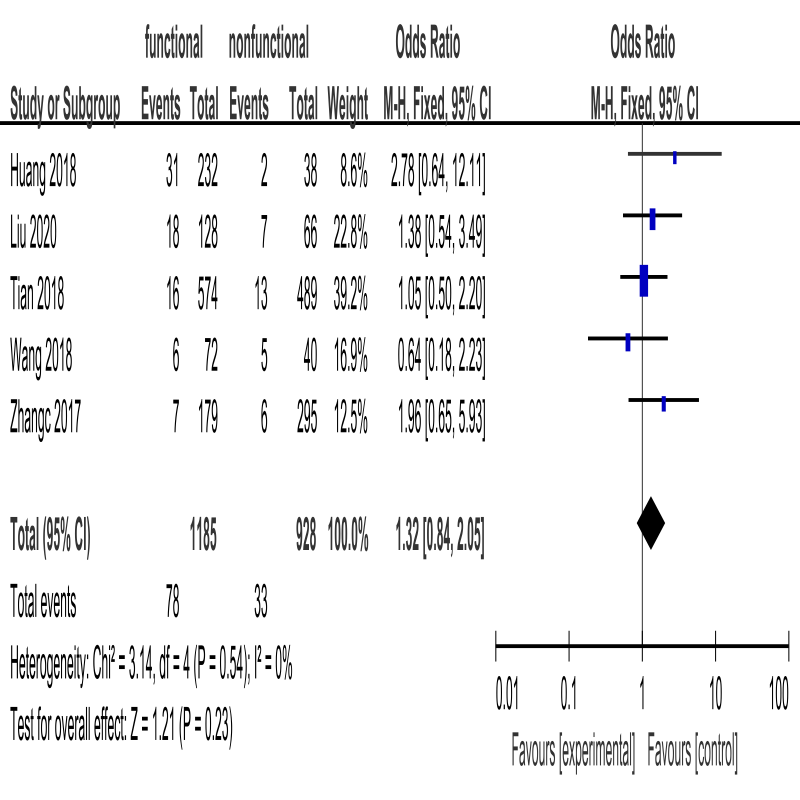


**Figure 8. Forest plot of postoperative CSF leakage according to functional adenoma type**

**Resection rate**

A total of six studies provided data regarding the relationship between resection rate and postoperative CSF leakage (Fig. 9). Meta-analysis suggested no significant difference in the incidence of postoperative CSF leakage between total resection and partial or subtotal resection (pooled OR: 0.77, 95% CI: 0.21–2.80). The sensitivity analysis suggested that when Xu’s study[14] was excluded, the result (pooled OR: 0.53, 95% CI: 0.14–1.99) was consistent with the overall result without excluding any studies. However, the heterogeneity remained high (I^2^ = 80%, *P* < 0.001).


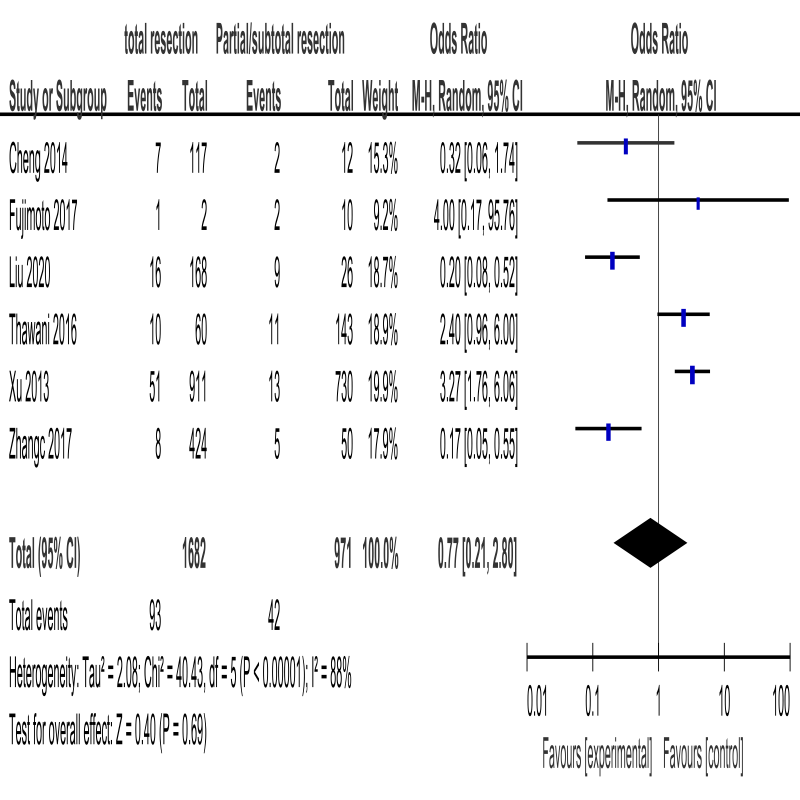


**Figure 9. Forest plot of postoperative CSF leakage according to resection rate**

**Perioperative lumbar drainage**

A total of five studies provided data regarding the relationship between perioperative lumbar drainage (LD) and postoperative CSF leakage (Fig. 10). Meta-analysis suggested that no significant differences in the incidence of postoperative CSF leakage between patients with LD and without LD (pooled OR: 0.61, 95% CI: 0.18–2.11). Sensitivity analysis suggested when Wang’s study[22] was excluded, the result (pooled OR: 0.36, 95% CI: 0.16–0.82) was different from the result without excluding any studies, and with no heterogeneity (I^2^ = 0%, P > 0.05). Through discussion with the researchers, Wang’s study[22] was determined to meet the inclusion criteria. Therefore, the credibility of this result was low.


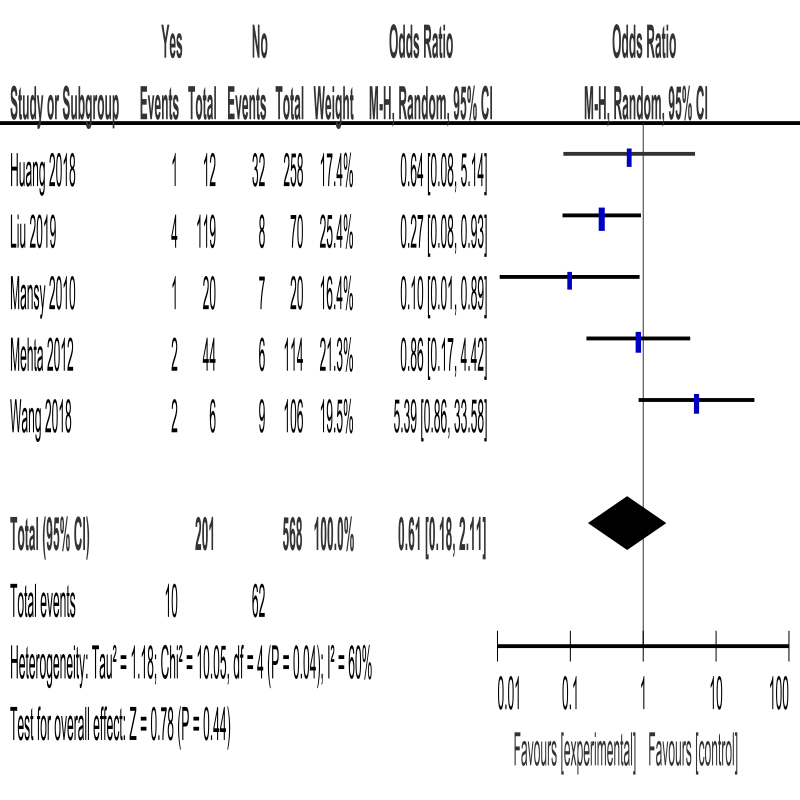


**Figure 10. Forest plot of postoperative CSF leakage according to perioperative lumbar drainage**
